# Triple-negative breast cancer: the importance of molecular and histologic subtyping, and recognition of low-grade variants

**DOI:** 10.1038/npjbcancer.2016.36

**Published:** 2016-11-16

**Authors:** Fresia Pareja, Felipe C Geyer, Caterina Marchiò, Kathleen A Burke, Britta Weigelt, Jorge S Reis-Filho

**Affiliations:** 1 Department of Pathology, Memorial Sloan Kettering Cancer Center, New York, NY, USA; 2 Department of Medical Sciences, University of Turin, Turin, Italy

## Abstract

Triple-negative breast cancers (TNBCs), defined by lack of expression of estrogen receptor, progesterone receptor and HER2, account for 12–17% of breast cancers and are clinically perceived as a discrete breast cancer subgroup. Nonetheless, TNBC has been shown to constitute a vastly heterogeneous disease encompassing a wide spectrum of entities with marked genetic, transcriptional, histological and clinical differences. Although most TNBCs are high-grade tumors, there are well-characterized low-grade TNBCs that have an indolent clinical course, whose natural history, molecular features and optimal therapy vastly differ from those of high-grade TNBCs. Secretory and adenoid cystic carcinomas are two histologic types of TNBCs underpinned by specific fusion genes; these tumors have an indolent clinical behavior and lack all of the cardinal molecular features of high-grade triple-negative disease. Recent studies of rare entities, including lesions once believed to constitute mere benign breast disease (e.g., microglandular adenosis), have resulted in the identification of potential precursors of TNBC and suggested the existence of a family of low-grade triple-negative lesions that, despite having low-grade morphology and indolent clinical behavior, have been shown to harbor the complex genomic landscape of common forms of TNBC, and may progress to high-grade disease. In this review, we describe the heterogeneity of TNBC and focus on the histologic and molecular features of low-grade forms of TNBC. Germane to addressing the challenges posed by the so-called triple-negative disease is the realization that TNBC is merely a descriptive term, and that low-grade types of TNBC may be driven by distinct sets of genetic alterations.

## Introduction

**Figure 1:**
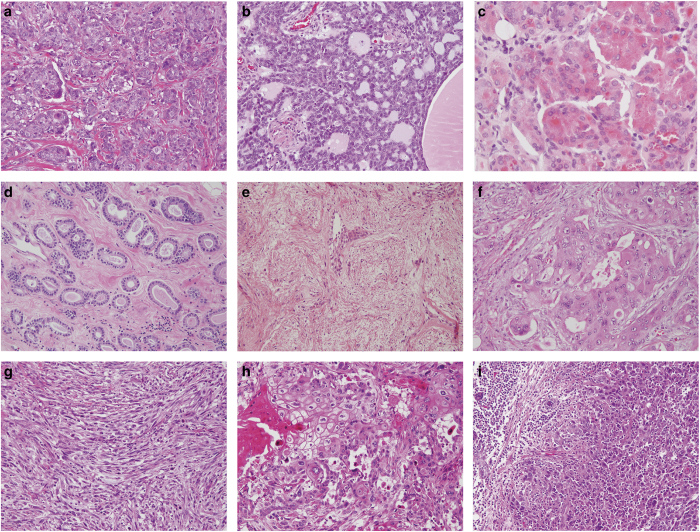
The spectrum of histologic subtypes of TNBCs and a non-obligate precursor of TNBC. (**a**) High-grade invasive ductal carcinoma of no special type, (**b**) low-grade adenoid cystic carcinoma, (**c**) low-grade acinic cell carcinoma, (**d**) microglandular adenosis, (**e**) low-grade metaplastic adenosquamous carcinoma, (**f**) high-grade apocrine carcinoma, (**g**) high-grade metaplastic spindle cell carcinoma, (**h**) high-grade metaplastic squamous cell carcinoma, and (**i**) high-grade carcinoma with medullary features. TNBC, triple-negative breast cancer.

Triple-negative (TN) breast cancers (TNBCs), defined by the lack of expression of estrogen receptor (ER), progesterone receptor (PR) and human epidermal growth factor receptor 2 (HER2), account for 12–17% of breast cancers.^1^ TNBCs have been shown to have a relatively aggressive clinical behavior, a high prevalence in women of Hispanic and African descent, an earlier age of presentation^2^ and a significant association with *BRCA1* germline mutations.^1^ As a group, TNBCs display a high risk of metastasis and death within 5 years after diagnosis.^3^ Nonetheless, TNBC is vastly heterogeneous and best considered as an umbrella term, encompassing a wide spectrum of entities with marked genetic, transcriptional, histological, and clinical differences.^4^ Although most TNBCs are of high grade and do display a relatively aggressive clinical behavior, there are forms of low-grade TN disease, which have been shown to have a more indolent behavior (Figure 1).^5–7^ In addition, recent studies have brought forth the existence of lesions initially thought to be mere benign breast conditions that likely constitute precursors of TNBCs.^8,9^

Here, we review the heterogeneity of TNBC and focus on the histologic and molecular features of low-grade forms of TNBC. The realization that TNBC is merely a descriptive term, and that low-grade types of TNBC may harbor distinct genetic alterations is central to the successful classification of these lesions into subtypes that are clinically meaningful and representative of the biology of the disease, and to the tailoring of therapies for patients with TNBC.

## Definition of TNBC

The term TNBC was first used in 2005 (ref. 10) to refer to a subset of breast cancer patients for whom chemotherapy was the only treatment available, given that patients with TN disease lack ER, PR, and HER2 and, therefore, are not eligible to receive hormonal therapy or anti-HER2 agents. From a scientific and translational research standpoint, the TN phenotype was also of interest, given that it was initially perceived as a potential surrogate for basal-like breast cancers, one of the ‘intrinsic gene’ subtypes of the disease characterized by lack of ER and HER2 mRNA expression, but expressing genes usually found in basal/ myoepithelial cells of the normal breast.^11^ Currently, TNBCs should be defined as invasive breast cancers lacking ER and PR expression and HER2 overexpression/*HER2* gene amplification according to the definitions put forward in the American Society of Clinical Oncology/College of American Pathologists guidelines for the assessment of ER, PR, and HER2,^12,13^ as these definitions refer to a subset of breast cancer patients who are not eligible for endocrine and anti-HER2 therapies.

## Genetic heterogeneity of TNBC

**Figure 2:**
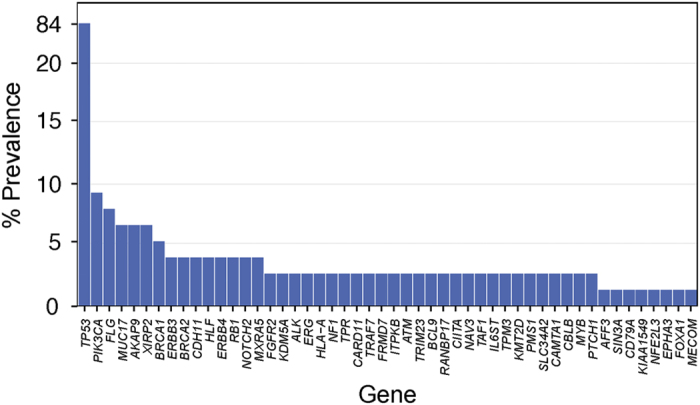
Somatic mutations affecting cancer genes in TNBCs from The Cancer Genome Atlas (TCGA). Bars indicate the frequency of somatic mutations affecting the 50 cancer genes most frequently mutated in TNBCs, based on a reanalysis of the 77 TNBCs from TCGA.^18^ Cancer genes are defined as per the cancer gene lists described by Kandoth *et al.*^14^ (127 significantly mutated genes), the Cancer Gene Census,^113^ or Lawrence *et al.*^114^ (Cancer5000-S gene set). TNBC, triple-negative breast cancer.

Given that the unifying feature of TNBCs is the lack of three biomarkers, the genomic heterogeneity of these tumors should not come as a surprise. As a group, TNBCs have been shown to be characterized by high levels of genetic instability, with a median of 1.7 (range 0.16–5.23) mutations/Mb,^14,15^ and complex patterns of copy number alterations (CNAs) and structural rearrangements.^16^ Unlike other forms of breast cancer, where several genes have been found to be mutated in >10% of cases, the only known cancer genes targeted by somatic mutations in 10% or more of TNBCs are *TP53* and *PIK3CA* (Figure 2). Importantly, however, TNBCs display a great variation in mutational content. Although some TNBCs harbor a limited number of somatic mutations, others display a high mutational burden affecting genes pertaining to numerous signaling pathways.^17^ In a way akin to ER-positive breast cancers, the most frequently mutated genes in TNBCs are *TP53* and *PIK3CA*, which are mutated in 82 and 10% of consecutive TNBCs, respectively.^18^ In contrast to ER-positive carcinomas, however, *TP53* somatic mutations in TNBCs are enriched for nonsense single-nucleotide variants and indels.^17,18^ Importantly, however, somatic mutations affecting other known cancer driver genes, including *PTEN*, *RB1*, *NF1*, *BRCA1, BRCA2, ERBB3*, *ERBB4*, *ALK*, are found in small subsets of TNBCs (Figure 2).^18^

The heterogeneity in mutational content observed in TNBCs is paralleled with an ample variation in their clonal composition, with the number of clones/subclones identified in a single tumor ranging from one to two per case, to multiple clones/subclones in some cases, underscoring a great variation in clonal evolution. *TP53*, *PIK3CA*, and *PTEN* mutations appear to be acquired early in tumorigenesis, whereas mutations involving cell motility and epithelial-to-mesenchymal transition genes display lower clonal frequencies, suggesting that those represent later events in tumor evolution.^17^ Importantly, in a substantial fraction of tumors, founder somatic mutations such as *TP53* and *PIK3CA* can be subclonal,^17^ providing evidence to suggest that a large subset of TNBCs are composed of mosaics of cancer cells at diagnosis.^17^

TNBCs, as a group, display complex patterns of CNAs, with multiples gains and losses intercalated across all the chromosomes and few focal high-level amplifications.^19^ Recurrent CNAs found in TNBCs include gains of 1q, 8q, and 10p, and losses of 5q and 8p, as well as *PARK2* intragenic deletions, *EGFR* and *FGFR2* amplifications, and *PTEN* loss.^17–21^ Notably, TNBCs lack concurrent 1q gains and 16q losses, changes typically found in ER-positive breast cancers.^22,23^

Functionally recurrent gene rearrangements have been described in TNBC. A subset of TNBCs harbor gene fusions involving Notch genes (*NOTCH1* and *NOTCH2*) and microtubule-associated serine-threonine kinase genes (*MAST1* and *MAST2*), which appear to be mutually exclusive.^24^ These findings might open new therapeutic avenues, as preclinical studies have shown that patient-derived xenograft models of Notch-altered tumors and cell lines harboring Notch fusion genes have been reported to be sensitive to Notch signaling inhibition.^24,25^ In addition, Banerji *et al.*^26^ reported on a recurrent *MAGI3*–*AKT3* fusion gene enriched in TNBC, which was detected in 7% of TNBC cases (5/72) by reverse transcription PCR (RT-PCR). A subsequent break-apart fluorescence *in situ* hybridization and RT-PCR study of 236 TNBCs failed to detect the *MAGI3*–*AKT3* fusion gene.^27^ Reanalysis of the cases reported by Banerji *et al.*^26^ to harbor the *MAGI3*–*AKT3* fusion gene using a hybrid capture array confirmed the presence of this fusion gene in a single TNBC, suggesting that the *AKT3*–*MAGI3* fusion gene is either a private genetic event or that its prevalence is substantially lower than that reported by Banerji *et al.*^26^

## Post-neoadjuvant chemotherapy of TNBC

Although neoadjuvant systemic therapy does not improve the overall survival of breast cancer patients compared with adjuvant chemotherapy, the achievement of pathologic complete response (pCR) following neoadjuvant chemotherapy is associated with an improved prognosis. TNBCs display the highest rates of pCR following neoadjuvant chemotherapy,^1,28–30^ with approximately 35–50% of TNBCs achieving pCR following anthracycline+taxane neoadjuvant chemotherapy regimens.^28,30^ Importantly, the patients with TNBC who achieve pCR have been shown to have an excellent long-term clinical outcome, with very few distant relapses; on the other hand, patients who have residual disease after neoadjuvant chemotherapy have a poor prognosis.^1,28–30^

A subset of TNBCs has been suggested to harbor homologous recombination DNA repair defects similar to those found in tumors arising in *BRCA1* and *BRCA2* mutation carriers.^31^ Given that tumors with homologous recombination DNA repair defects may show greater sensitivity to agents that cause DNA double-strand breaks and crosslinks,^32,33^ such as platinum salts and inhibitors of the Poly(ADP) Ribose Polymerase (PARP), it has been posited that, as a group, patients with TNBCs may benefit from platinum-based chemotherapy. There is burgeoning evidence to demonstrate that a subset of TNBC patients may benefit from the addition of platinum-based chemotherapy to current chemotherapy regimens. Randomized prospective clinical trials (CALGB 40603 and GeparSixto)^30,34^ have demonstrated that the addition of carboplatin to doxorubicin and paclitaxel in patients with TNBC results in significantly higher rates of pCR than the current anthracycline+taxane-based chemotherapy (pCR rates 60% (54–66%) vs. 46% (40–53%) in the CALGB 40603 trial^34^ and 53.2% (54.4–60.9%) vs. 36.9% (29.4–44.5%) in the GeparSixto trial^30^). Although the concept of BRCAness is known for over a decade,^31^ biomarkers to define which TNBC patients are likely to benefit from this regimen have yet to be fully developed.^35^

Given that TNBC patients with residual disease after neoadjuvant chemotherapy have a shorter overall survival than patients with non-TN breast cancers,^36^ the identification of targetable alterations in TN residual disease is of paramount importance. Balko *et al.*^37^ have recently analyzed a series of 74 residual TNBCs following neoadjuvant chemotherapy and showed that >90% of cases had alterations in at least one clinically targetable pathway (*PTEN*, *JAK2*, *CDK6*, *CCND1*, *CCND2*, *CCND3* and *IGF1R*). In addition, a higher frequency of potentially targetable alterations was detected in post-treatment TNBCs compared with primary basal-like breast cancers from TCGA. A frequent *MYC* and *MCL1* co-amplification in residual TNBCs following neoadjuvant chemotherapy was detected, with *MCL1* gains in 83% of *MYC*-amplified cases. Moreover, concurrent forced expression of *MYC* and *MCL1* in MCF10A cells enhanced cellular colony formation, whereas their silencing increased cellular sensitivity to doxorubicin.^37^

The same group has also identified in TNBCs following neoadjuvant chemotherapy downregulation of *DUSP4,* a phosphatase that negatively regulates members of the MAP-kinase pathways.^38^ Reduced expression of *DUSP4* was found to be associated with a worse outcome in TNBC patients,^39^ and when detected in TNBCs after neoadjuvant therapy DUSP6 reduced expression was associated with treatment-refractory high Ki67 scores and shorter recurrence-free survival.^38^ In addition, MEK inhibition synergized with docetaxel in TNBC xenografts,^38^ suggesting that this therapeutic combination might potentially benefit TNBC patients with residual disease after neoadjuvant chemotherapy.

Taken together, neoadjuvant therapy of patients with TNBC has revealed that a subset of these cancers is remarkably sensitive to conventional cytotoxic agents and that this effect is increased by the addition of platinum salts. Opportunities for translational research in this area include the developments of biomarkers to predict pCR in patients with TNBC, the analysis of post-neoadjuvant chemotherapy residual disease, and whether this residual disease differs from (micro)metastatic disease in these patients.

## Transcriptomic heterogeneity of TNBC

Albeit initially perceived as a synonym with basal-like breast cancer at the transcriptomic level, several studies have demonstrated that TNBCs display a great deal of heterogeneity and that these two definitions are not synonymous.^40^ An additional intrinsic subtype preferentially of TN phenotype was described subsequently, namely claudin-low, which expresses low levels of luminal differentiation markers, is enriched for the expression of epithelial-to-mesenchymal transition, immune response and cancer stem cell-related genes.^41^

**Figure 3:**
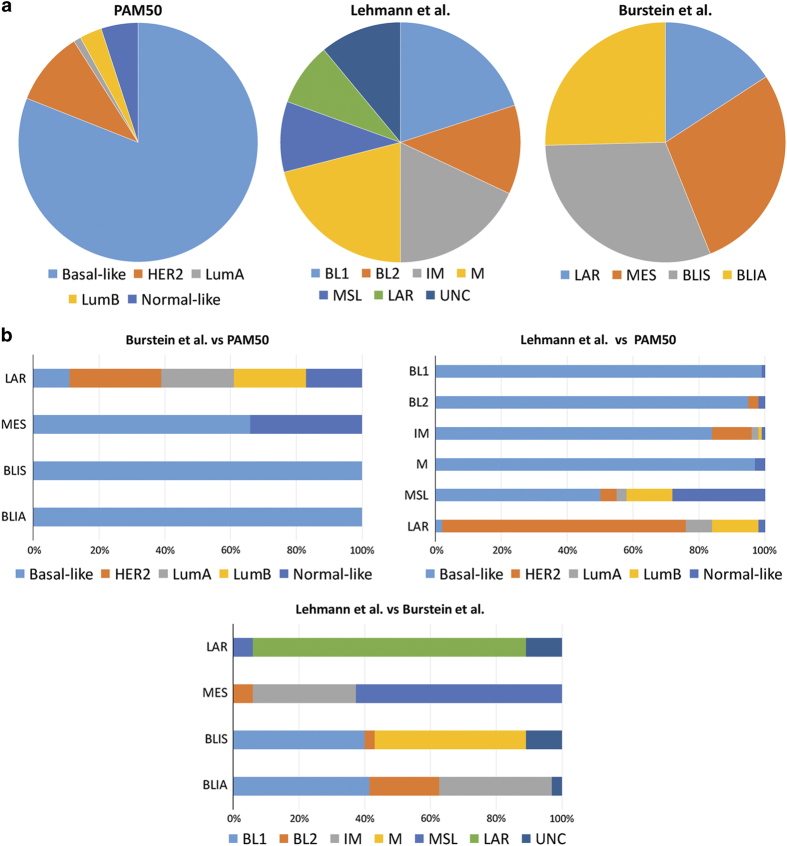
Comparative analysis of molecular subclassification systems applied to TNBCs. (**a**) Prevalence of each subtype according to PAM50,^43^ Lehmann *et al.*,^43^ and Burstein *et al.*^47^ (**b**) Comparison between the distinct classifications; Burstein *et al.* versus PAM50,^47^ Lehmann *et al.* versus PAM50,^43^ and Burstein *et al.* versus Lehmann *et al.*^47^ BL1, basal-like 1; BL2, basal-like 2; BLIA, basal-like immune-activated; BLIS, basal-like immunosuppressed; IM; immunomodulatory; LAR, luminal androgen receptor; LumA, luminal A; LumB, luminal B; M, mesenchymal; MES, mesenchymal; MSL, mesenchymal stem-like; TNBC, triple-negative breast cancer; UNC, unclassified.

The seminal studies carried out by Lehmann *et al.*^42,43^ further demonstrated the transcriptional heterogeneity of TNBCs, revealing the existence of six subtypes of TN disease: basal-like 1, basal-like 2, immunomodulatory, mesenchymal, mesenchymal stem-like and luminal androgen receptor (LAR; Figure 3). Among the basal-like subtypes, the basal-like 1 subset was found to be enriched in cell division and DNA damage response pathways, whereas the basal-like 2 group displayed an association with growth factor signaling and myoepithelial marker expression. The immunomodulatory subtype is characterized by immune cell processes and immune signaling cascades. Although the mesenchymal stem-like and mesenchymal subtypes share several transcriptomic similarities and are enriched for genes implicated in cell motility and epithelial-to-mesenchymal transition, the mesenchymal stem-like subtype displays lower expression of genes associated with cellular proliferation, and is enriched for genes related to mesenchymal stem cells. The LAR subtype displays a luminal-like gene expression pattern despite ER-negativity, most likely due to androgen receptor activation. Comparative analyses of these six subtypes with the intrinsic gene subtypes (Figure 3b) revealed that basal-like 1, basal-like 2, immunomodulatory and mesenchymal TNBCs are preferentially of basal-like intrinsic subtype, that a large proportion of mesenchymal stem-like TNBCs fit the intrinsic normal-like or claudin-low^43,44^ subtypes, and that the LAR subgroup corresponds in most part to the rare TNBCs classified by PAM50 as luminal or HER2-enriched.^42,43^ It should be noted that this six TNBC subtype classification may have therapeutic implications, given that (i) xenografts of breast cancer cell lines classified as of basal-like subtypes were found to be sensitive to platinum salts, whereas mesenchymal and LAR subtype xenografts were sensitive to PI3K/mTOR pathway inhibition and anti-androgen therapy, respectively;^42^ and (ii) that approximately 50% of patients with basal-like 1 TNBCs were reported to evolve to pCR following standard neoadjuvant chemotherapy, whereas the pCR rates for other subgroups, such as LAR (10%) and basal-like 2 (0%), were found to be markedly lower.^45^

Curtis *et al.*^23^ proposed an alternative taxonomy for breast cancer based on the integration of CNAs and gene expression profiles, which defined 10 integrative clusters (IntClust 1–10). IntClust 10 is composed mainly by poorly differentiated TNBCs, with highly recurrent *TP53* mutations and intermediate genomic instability, and is characterized by poor prognosis in the first 5 years after diagnosis. On the other hand, approximately a fourth of all TNBCs correspond to the IntClust 4 subtype, which has low levels of genomic instability, absence of CNAs, marked lymphocytic infiltrate and a better outcome,^46^ providing another level of evidence to demonstrate the genomic heterogeneity of TNBCs.

More recently, Burstein *et al.*^47^ put forward yet another gene expression classification, which categorizes TNBCs in luminal/androgen receptor (LAR), mesenchymal (MES), basal-like/immune-suppressed (BLIS), and basal-like/immune activated (BLIA) subtypes (Figure 3), which have distinct clinical outcomes. The BLIS subset displays the best outcome, whereas the BLIA subgroup confers the poorest prognosis.^47^ Moreover, subtype-specific gene amplifications were detected. LAR, MES, BLIS, BLIA subtypes harbor amplifications of *CCND1*, *EGFR, FGFR2*, and *CDK1*, respectively. Although ‘Burstein’s’ and ‘Lehmann’s’ LAR and mesenchymal subtypes showed significant overlap, Burstein’s BLIS and BLIA subtypes were a mixture of Lehmann’s groups^47^ (Figure 3b), suggesting that not all TNBC gene expression subtypes are stable and reproducibly identified, as previously demonstrated for the intrinsic gene subtype classification.^48^

## The immune MILIEU as a prognostic and predictive factor for TNBC

Gene expression profiling has allowed significant progress in our ability to assess prognosis and predict response to therapy in breast cancer.^49^ First-generation prognostic signatures,^50^ which rely markedly on proliferation-related genes, allow reliable stratification of ER-positive breast cancer, however these have a limited utility in TNBC, mainly due to the high proliferative nature of most ER-negative cancers, and, therefore, the lack of discriminatory power of first-generation prognostic signatures in triple-negative disease. Activation of immune response genes was shown, however, to be a good prognostic factor in ER-negative cancers.^51,52^ Second-generation signatures based on immune response-related genes have been developed;^53,54^ although these signatures allow for the stratification of TNBC patients according to overall and relapse-free survival, their clinical utility remains negligible owing to the high number of events in this population, even in patients with favorable signatures.^49^

Along the same lines, the relevance of the tumor-infiltrating lymphocytes (TILs) as a prognostic and predictive biomarker in TNBC is increasingly appreciated. In 2010, Denkert *et al.*^55^ described a quantitative assessment of TILs as a predictor of response to neoadjuvant chemotherapy. Subsequent retrospective analyses of clinical trials have confirmed that the levels of TILs are predictive of pCR and increased disease-free or overall survival,^56,57^ and guidelines for evaluation of TILs in breast cancer were provided by the International TIL Working Group.^58^ Although the analytical validity and reproducibility of those guidelines have been demonstrated in several studies,^59,60^ the clinical utility of TILs assessment, in a way akin to the second-generation signatures, remains limited given that TIL levels are prognostic in TNBC patients treated with chemotherapy and robust evidence for changing practice according to the levels of TILs has yet to be provided.

A recent gene expression analysis of immune activating and immunosuppressive factors in TNBC and HER2-positive carcinomas of the GeparSixto study unveiled three subgroups of tumors, immune group A, B, and C with low, intermediate, and high immune gene expression levels, respectively. Noticeably, tumors belonging to the immune group C had a higher extent of TIL infiltrate and better pCR rates than those of immune groups A and B.^61^ Moreover, the extent of lymphocytic infiltrate in residual TNBC following neoadjuvant chemotherapy has been associated with metastasis-free and overall survival, highlighting the potential of this biomarker in the post-neoadjuvant setting to identify patients at risk of relapse.^62^

## TNBCs: the importance of histologic subtyping

The large majority of TNBCs are high-grade invasive carcinomas of no special type displaying pushing invasive borders, central necrosis, brisk lymphocytic infiltrates, marked nuclear pleomorphism, and numerous mitoses.^1,4^ Nevertheless, there is a multitude of rare histologic special types of breast cancer that are consistently of TN phenotype (Figure 1).

Some high-grade special histologic types of breast cancer, including carcinomas with apocrine features, carcinomas with medullary features, and metaplastic breast carcinomas (MBCs) almost invariably display a TN phenotype.^63^ Notably, among TNBCs, carcinomas with apocrine features are the ones most likely to express androgen receptor and display a molecular apocrine or LAR gene expression profile.^64^ Thus, their identification may suggest potential sensitivity to anti-androgen receptor agents and may trigger androgen receptor testing, but does not carry definite prognostic information as their outcome is uncertain and has been reported to be comparable to that of conventional invasive carcinomas of no special type.^65^ Likewise, contradictory data have been published regarding the prognostic impact of androgen receptor expression in TNBCs.^66,67^

Medullary carcinoma is a controversial histologic special type of breast cancer, which has been reclassified as a histologic pattern (i.e., carcinomas with medullary features) in the latest World Health Organization classification.^68^ Well-circumscribed borders, a syncytial growth pattern, and brisk lymphocytic infiltrate are the hallmark features of the so-called medullary carcinoma; their histologic identification, however, has been shown to lack in inter-observer reproducibility. Despite worrisome cytological features and high mitotic activity, carcinomas with medullary features are historically perceived to have an excellent outcome.^69^ Given the low inter-observer agreement rate for the identification of this histologic subtype, a diagnosis of carcinoma with medullary features does not carry any therapeutic implication and patients with these cancers should be treated following the same protocols for common forms of TNBC. In fact, one could argue that the good prognosis historically reported to medullary carcinomas is merely a reflection of the brisk lymphocytic infiltrate that these tumors display, which has now been validated by level I evidence as a prognostic marker for patients with TNBC treated with chemotherapy.^56,57^

MBCs encompass a spectrum of tumors with squamous and/or mesenchymal differentiation,^70^ are mostly high-grade lesions, display worse outcome than conventional TNBCs,^71^ and show significant inter- and intra-tumor heterogeneity.^72,73^ These tumors are preferentially classified as of claudin-low or basal-like intrinsic subtype,^73–75^ however, there is evidence that their histologic subtype, as well as the subtype present in the sample subjected to molecular analysis, have an impact on their genomic profile.^73^ The spindle cell MBCs are preferentially of claudin-low intrinsic subtype, whereas the squamous and chondroid MBCs are classified more frequently as basal-like.^73^ At the genetic level, MBCs are enriched for genetic alterations affecting Wnt and PI3K pathways,^75,76^ in particular in the form of *PIK3CA* mutations. The data on the repertoire of genetic alterations in MBCs is scarce, however, our group has demonstrated that histologically distinct morphological components within individual MBCs may display distinct patterns of CNAs, despite being clonally related.^72^

**Figure 4:**
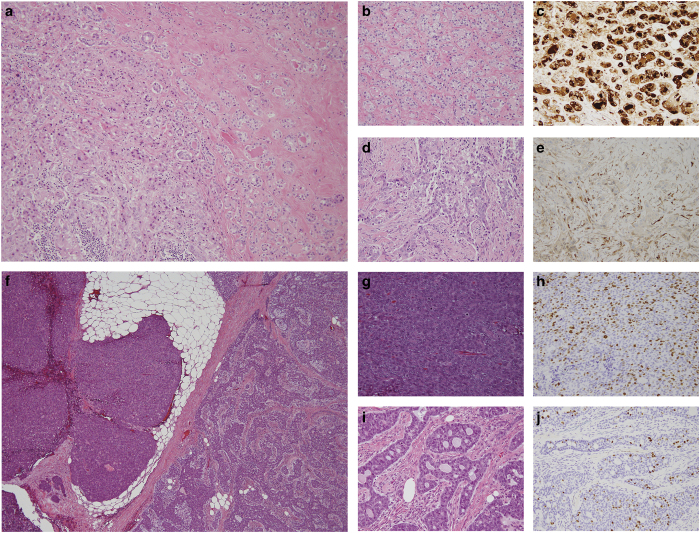
Progression from low- to high-grade within the proposed low-grade triple-negative breast neoplasia family and salivary gland-like tumors of the breast. (**a**) Representative low-power magnification of a high-grade invasive carcinoma of no special type (left) arising in microglandular adenosis (right). (**b**) Representative high-power magnification of microglandular adenosis with (**c**) diffuse immunohistochemical expression of lysozyme, a marker of serous acinar differentiation. (**d**) Representative high-power magnification of associated high-grade invasive carcinoma of no special type, with (**e**) focal lysozyme expression by immunohistochemistry. (**f**) Representative low-power magnification of a high-grade invasive carcinoma of no special type (left) arising in an adenoid cystic carcinoma (right). (**g**) Representative high-power magnification of a high-grade invasive carcinoma of no special type with (**h**) high Ki67 proliferation rate. (**i**) Representative high-power magnification of associated adenoid cystic carcinoma with (**j**) low Ki67 proliferation rate.

**Figure 5:**
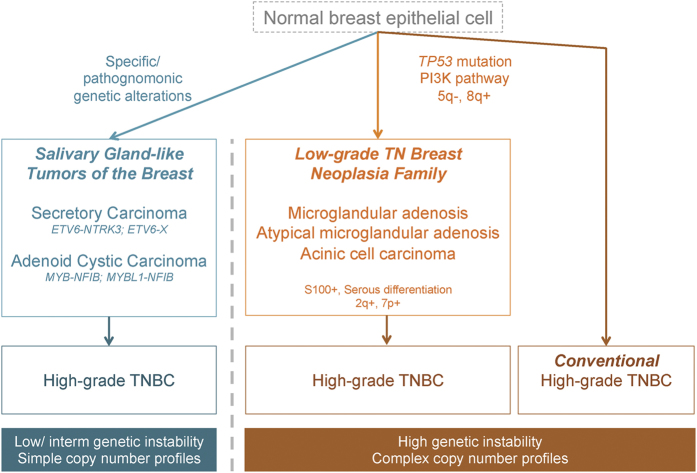
Hypothetical model of potential evolutionary paths of TNBCs. We propose that in addition to high-grade TNBCs, two subtypes of low-grade TNBCs can be identified on the basis of their distinctive histology and molecular features: salivary gland-like tumors of the breast, which are underpinned by specific/pathognomonic genetic alterations, and the proposed low-grade breast triple-negative neoplasia family, whose tumors display genomic profiles similar to those of conventional high-grade TNBCs. Please note that both low-grade subgroups can progress to high-grade TNBCs, however, the high-grade TNBCs arising in salivary gland-like tumors also differ from conventional TNBCs at the genetic level and harbor the same genetic aberrations identified in their respective low-grade counterparts. TNBC, triple-negative breast cancer.

Although as a group TNBCs display an aggressive clinical behavior, a subset of these cancers are characterized by low histologic grade and an indolent behavior. For example, even among MBCs, low-grade variants exist, such as the low-grade spindle and adenosquamous carcinomas, which display a less aggressive clinical course.^68^ Among low-grade TN neoplasms, at least two subsets can be distinguished: (i) carcinomas with salivary gland-like morphology, which are underpinned by specific/pathognomonic genetic alterations and display low-to-intermediate levels of genetic instability; (ii) a subgroup of low-grade lesions, including lesions once considered to be benign hyperplastic proliferations (i.e., microglandular adenosis (MGA)), atypical lesions (i.e., atypical microglandular adenosis (AMGA)) and invasive carcinomas (i.e., acinic cell carcinoma (ACC)), that, despite their low-grade morphology and good outcome, recapitulate the complex genomic landscape of usual TNBCs (Figures 4 and 5).

### Salivary gland-like tumors of the breast

This group of TNBCs recapitulate neoplasms primary of the salivary glands not only morphologically, but also genetically. In contrast to conventional TNBCs, these tumors lack recurrent *TP53* aberrations, display few copy number alterations and harbor specific/pathognomonic genetic alterations. This group includes the well-characterized adenoid cystic carcinoma (AdCC) and secretory carcinoma, underpinned by *MYB*–*NFIB* and *ETV6*–*NTRK3* fusion genes, respectively.^77,78^ Additional lesions rarely occurring in the breast, yet comprehensively studied when arising in the salivary glands, can tentatively be included in this subgroup, such as the polymorphous low-grade adenocarcinoma and mucoepidermoid carcinoma, which are characterized by *PRKD1* hotspot mutations^79^ and *MAML2* rearrangements,^80^ respectively. Adenomyoepitheliomas of the breast, though heterogeneous, are frequently of TN phenotype and can be morphologically identical to epithelial–myoepithelial carcinomas of the salivary glands. Recent data suggest that their phenotypic similarities may be underpinned by a similar constellation of mutations (Reis-Filho *et al.*, manuscript under review).^81^

*Adenoid cystic carcinoma.* AdCCs, albeit originally described in the salivary glands, can also arise in other anatomical sites, including the lungs and breast. Breast AdCCs account for less than 0.1% of breast carcinomas, and typically show a good prognosis, in contrast to the poor long-term outcomes in head and neck AdCCs.^82^ AdCCs are composed of a dual population of luminal and myoepithelial/basal cells, growing in cribriform, tubular and/or solid patterns. The vast majority (>95%) is TN^82^ and at the transcriptomic level they pertain to the basal-like subtype.^83^ No data are available in regards to AdCC and the TNBC gene expression classification by Lehmann *et al.*^42^

Regardless of its anatomic location, the hallmark genetic alteration of AdCC is a rearrangement of the *MYB* gene, most frequently in the form of *MYB*–*NFIB* fusion gene, resulting in the *t*(6;9)(q22–23;p23–24) translocation.^77^ The prevalence of such alteration ranges from 23 to 100% in breast AdCCs. Notably, breast AdCCs lacking the *MYB–NFIB* rearrangement have been shown to be morphologically similar to those harboring this fusion gene.^84^ Distinct rearrangements affecting a second MYB family gene (*MYBL1*) have been demonstrated in AdCCs of other sites,^85^ acting in remarkably similar ways, and theoretically also detectable in breast AdCCs.

In contrast to common forms of TNBCs, AdCCs display quiet genomes lacking high-level amplifications or homozygous deletions,^84^ as well as CNAs frequently present in usual TNBCs, such as 8q gain and 5q loss. Breast AdCCs rather show recurrent 17q21-q25.1 gains and 12q12-q14.1 losses.^19,84^ Interestingly, AdCCs occurring in the salivary gland also display 12q13 losses.^86^ Similar to AdCCs of the salivary glands, the mutation rate of breast AdCCs is low.^84^ They lack *TP53* and *PIK3CA* somatic mutations and preferentially harbor mutations affecting genes associated with chromatin remodeling, cell adhesion and signaling cascades.^84^ Breast AdCCs have also been shown to display recurrent mutations in *TLN2*, *MYB*, and *BRAF*^84,87^ and to harbor mutations in cancer genes reported to be mutated in salivary glands AdCCs,^86^ such as *SF3B1*, *FBXW7*, and *FGFR2*.

High-grade transformation has been described in breast and salivary gland AdCCs^7,88,89^ (Figures 4f and g). Notably, high-grade TNBCs arising in low-grade AdCCs^7^ or high-grade basaloid AdCCs^90^ may also harbor the *MYB*–*NFIB* fusion gene. Few studies suggested that in salivary glands p53 (ref. 89) or PTEN^91^ inactivation, or yet *MYC* amplification^88^ may have a role in this phenomenon. Our group has recently reported on two breast AdCCs with high-grade transformation; our findings confirmed that progression occurred via the acquisition of additional genetic events and/or clonal selection; however, none of the genetic alterations reported in the progression of salivary gland AdCCs were found in breast AdCCs.^7^ In a distinct study of a single breast AdCC metastasizing to the kidney,^92^ *PIK3CA* and *PTEN* mutations were found in both the primary and metastatic tumors, but, the metastatic deposits showed increased *PTEN* promoter methylation and lower *PTEN* gene expression levels. It seems therefore unlikely that a single genetic event is responsible for the high-grade transformation observed in human AdCCs.

*Secretory carcinoma.* Secretory carcinoma is a rare entity, accounting for less than 0.15% of breast cancers.^68^ Although initially described in children and named ‘juvenile carcinoma’, it was later shown to occur at a median age of 53 years.^93^ This entity has an excellent clinical outcome, with protracted survival even in the presence of nodal involvement and metastatic disease.^93^ Morphologically it displays tubular, solid and/or microcystic growth patterns with intra- and extra-cellular dense eosinophilic secretions. Although the vast majority of cases are low-grade TNBCs, cases of high-grade or with weak hormone receptor expression have been reported.^6^

Over 90% of secretory carcinomas harbor the *t*(12;15)(p13;q25) translocation resulting in the *ETV6*–*NTRK3* fusion gene.^78^ Although this translocation also underpins a variety of neoplasms of other sites (i.e., infantile fibrosarcoma, cellular congenital mesoblastic nephroma, acute myelogenous leukemia), in the context of breast carcinomas it is pathognomonic of secretory carcinoma.^94^ Importantly, the ETV6–NTRK3 fusion protein can be inhibited by crizotinib^95^ and other small molecule inhibitors, potentially offering a therapeutic strategy for the rare cases of metastatic and chemoresistant breast secretory carcinomas.^6^

The salivary gland counterpart of breast secretory carcinoma was first recognized due to the discovery that *ETV6*–*NTRK3* translocation underpins lesions morphologically similar to breast secretory carcinomas but previously classified as unusual variants of salivary gland ACCs.^96,97^ These lesions were then renamed mammary analog secretory carcinoma.^96^ A later study found that these tumors may harbor *ETV6* rearrangements with an unknown partner (*ETV6*-*X*),^98^ which theoretically may also occur in the breast counterpart.

Secretory carcinomas have simple genomes with few CNAs.^6,99^ Recurrent 8q, 1q, 16pq, and 12p gains, along with 22q losses have been identified.^6,99,100^ Del Castillo *et al.*^6^ have reported on a case of a lethal high-grade secretory carcinoma with fluorescence *in situ* hybridization-proven *ETV6* rearrangement, which despite harboring a simple genome, displayed more gains and losses of entire chromosomes and chromosomal arms than lower-grade tumors. Further studies of secretory carcinomas with high-grade transformation are warranted to define their prognosis and molecular underpinning.

### Low-grade TN breast neoplasia family

It is currently perceived that breast cancer evolution can be stratified into two main pathways according to ER pathway activation.^101^ ER-positive breast neoplasms encompass a spectrum of pre-invasive (columnar cell lesions, atypical ductal hyperplasia, lobular neoplasia, and low-grade ductal carcinoma *in situ*) and invasive lesions (invasive tubular, lobular, and low-grade ductal carcinomas), and progression from low- to high-grade lesions may take place.^22^ Owing to their frequent coexistence and similar pattern of genetic alterations (e.g., *PIK3CA* mutations and deletions of 16q and gains of 1q^23,101^), low-grade ER-positive non-obligate precursors and invasive carcinomas have been grouped together under the term ‘low-grade breast neoplasia family’.^101^

Akin to the low-grade ER-positive breast neoplasia family, a subset of related low-grade neoplastic entities can also be distinguished among the TN lesions of the breast. Indeed, MGA, AMGA, and ACC show overlapping morphology and immunophenotype (lack of ER, PR, and HER2 and expression of S100 protein), and are characterized by nearly indistinguishable genomic landscapes to those of common forms of TNBC.^8,9,102–106^ When not associated with high-grade TNBCs, these lesions have an indolent clinical behavior and limited metastatic potential despite the worrisome genomic landscape, and should be managed accordingly. Progression to high-grade TNBCs, however, is not uncommon.^102^ Notably, in both ACC and MGA/AMGA, the development of metaplastic TNBC has been reported by independent investigators.^9,105^ We can therefore hypothesize within the ER-negative branch of breast cancer evolution the existence of a ‘low-grade TN breast neoplasia family’ comprising MGA, AMGA, and ACC, which may give rise to *bona fide* high-grade TNBCs (Figures 4a–e).^106^

### Microglandular adenosis

MGA is histologically characterized by a haphazard proliferation of small glands infiltrating adipose and collagenous tissue, without eliciting a desmoplastic reaction.^68^ Although surrounded by a basement membrane, MGA acini lack a myoepithelial cell layer, in a way akin to invasive carcinomas. Although some have regarded MGA as a benign hyperplastic lesion,^103^ it is currently recognized that MGA encompasses a spectrum of lesions including pure MGA without atypia, atypical MGA (AMGA), and MGA associated with invasive carcinoma.^8,9,102–106^ Notably, MGA, AMGA, and associated high-grade TNBCs display a similar phenotype, including the expression of S100 protein, and pattern of genetic alterations.^8,9,104–106^ These findings are consistent with the notion that MGA/AMGA are in fact clonal neoplastic lesions and non-obligate precursors of TNBCs.

As a group, MGA/AMGA display complex copy number profiles with recurrent 5q losses and 8q gains.^9,104,105^ Massively parallel sequencing analysis has revealed that these lesions harbor highly recurrent (~80%) *TP53* mutations and a vast repertoire of mutated genes at low frequency, including *BRCA1*, PI3K pathway genes (*PTEN*, *PIK3CA*, and *INPP4B*) and genes encoding for receptor tyrosine kinases (*ERBB3*, *FGFR2*).^9^ Significant heterogeneity, however, is observed. Current data favor that the majority of pure MGAs differ from carcinoma-associated MGA/AMGA, given that pure MGAs lack *TP53* mutations and copy number alterations affecting genomic regions commonly altered in TNBCs. It is therefore possible that acquisition of *TP53* mutations is a driver of progression of MGAs and that the early genetic alterations responsible for the development of these lesions have yet to be unveiled.^9^

### Acinic cell carcinoma

ACC grows in microglandular and nested patterns and is defined by the presence of diffuse serous differentiation featuring fine cytoplasmic zymogen-type granules, reminiscent of the acinic cells in the salivary glands.^107^ Notably, serous differentiation is also observed in MGA and MGA-associated carcinomas.^5,106^ ACCs have a good prognosis, with infrequent local and distal recurrences.^68^ The latter are usually associated with the presence of a high-grade component.^108^ In fact, in a way akin to MGA, progression to a high-grade TNBC of distinct histologic type may not be a rare event. In our series of eight ACCs, six were admixed with a high-grade non-acinic cell component.^5^

Unlike other low-grade TNBCs that resemble their salivary gland counterparts at the genomic level, ACCs arising in the breast and in the salivary glands appear to be different entities, with distinct histologic features and genomic alterations.^107,109^ At the molecular level, breast ACCs harbor a complex pattern of CNAs, with recurrent gains of 1q, 2q, and 8q and losses of 3p, 5q, 12q, 13q, 14q, 17p, and 17q.^5^ In addition, they display a high mutational burden with *TP53* and *PIK3CA* mutations found in 80 and 10% of cases, respectively. This is in stark contrast to salivary gland ACCs that lack mutations affecting these genes.^5,109^ In addition, somatic mutations of other genes altered in common forms of TNBC, such as *MTOR*, *CTNNB1*, *BRCA1*, *ERBB3*, *INPP4B*, and *FGFR2*, have also been observed in breast ACCs.^5^

Akin to common forms of TNBC,^110^ an association between ACC and *BRCA1* inactivation has been documented. A breast ACC has been reported in a *BRCA1* mutation carrier; the tumor harbored a *BRCA1* loss of heterozygozity coupled with a *TP53* somatic mutation.^111^ We have reported on an ACC with somatic *BRCA1* loss of heterozygozity and *TP53* mutation, in the background of a germline frameshift *BRCA1* mutation, and an additional case with a *BRCA1* somatic truncating mutation with loss of heterozygozity of the *BRCA1* wild-type allele and *TP53* somatic mutation.^5^ Finally, we have also observed a case of MGA-associated carcinoma with similar somatic alterations affecting *BRCA1* and *TP53*,^9^ as well as MGA lesions in *BRCA1* mutation carriers (Geyer & Reis-Filho, unpublished data). Somatic abrogation of *BRCA1* and *TP53* in conditional mouse models usually gives rise to high-grade breast tumors; nonetheless, some mice may develop well-differentiated tumors.^112^ Taken together, these findings support the contention that the concurrent loss of function of *BRCA1* and *TP53* does not necessarily result in the development of high-grade breast cancers.

## Conclusion

TNBC is an operational term that defines a wide spectrum of entities with different biology and clinical behavior. Although most TNBCs are high-grade tumors with a relatively poor prognosis, a subset of low-grade TNBCs displays a favorable outcome. Low-grade TNBCs can be further classified in at least two subgroups. The first comprises the heterogeneous group of salivary gland-like tumors of the breast, where each entity is underpinned by specific genetic fusion genes or hotspot mutations, and lack the genomic features of common forms of TNBCs. The second group, the so-called low-grade TN breast neoplasia family, encompasses MGA, AMGA, and ACC, which are phenotypically similar and recapitulate the genetic alterations found in conventional TNBCs. Progression from low- to high-grade lesions may occur in both subgroups (Figures 4 and 5), though likely at a higher rate within the second group. Notably, high-grade TNBCs arising in salivary gland-like tumors are genetically more similar to their low-grade counterparts than to conventional TNBCs and the high-grade TNBCs in this context also harbor the pathognomonic genetic alteration that characterizes the low-grade lesion. Despite additional methods to stratify TNBCs into clinically meaningful subtypes being on record,^42,43^ histologic subtyping of these tumors is not a mere academic exercise, given that the therapeutic approaches for the rare low-grade subtypes of TNBC fundamentally differ from those of high-grade TNBCs.
